# Analysis of Macronutrients in Soil Using Impedimetric Multisensor Arrays

**DOI:** 10.1021/acsomega.4c04452

**Published:** 2024-07-25

**Authors:** Maria Luisa Braunger, Mario Popolin Neto, Dmitry Kirsanov, Igor Fier, Lucas R. Amaral, Flavio M. Shimizu, Daniel S. Correa, Fernando V. Paulovich, Andrey Legin, Osvaldo N. Oliveira, Antonio Riul

**Affiliations:** † Instituto de Física “Gleb Wataghin” (IFGW), Universidade Estadual de Campinas—UNICAMP, Campinas 13083-859, São Paulo, Brazil; ‡ Federal Institute of São Paulo—IFSP, Araraquara 14804-296, São Paulo, Brazil; § Institute of Chemistry, Mendeleev Center, St. Petersburg State University, Universitetskaya nab.7/9, St. Petersburg 199034, Russia; ∥ Laboratory of Artificial Sensory Systems, ITMO University, Kronverkskiy pr, 49, St. Petersburg 197101, Russia; ⊥ Quantum Design Latin America, Campinas 13080-655, São Paulo, Brazil; # School of Agricultural Engineering (FEAGRI), University of Campinas—UNICAMP, Campinas 13083-875, São Paulo, Brazil; ¶ Nanotechnology National Laboratory for Agriculture (LNNA), Embrapa Instrumentação, São Carlos 13560-970, São Paulo, Brazil; ∇ Department of Mathematics and Computer Science, Eindhoven University of Technology (TU/e), Eindhoven 5600 MB, The Netherlands; ○ São Carlos Institute of Physics (IFSC), University of São Paulo—USP, São Carlos 13566-590, São Paulo, Brazil

## Abstract

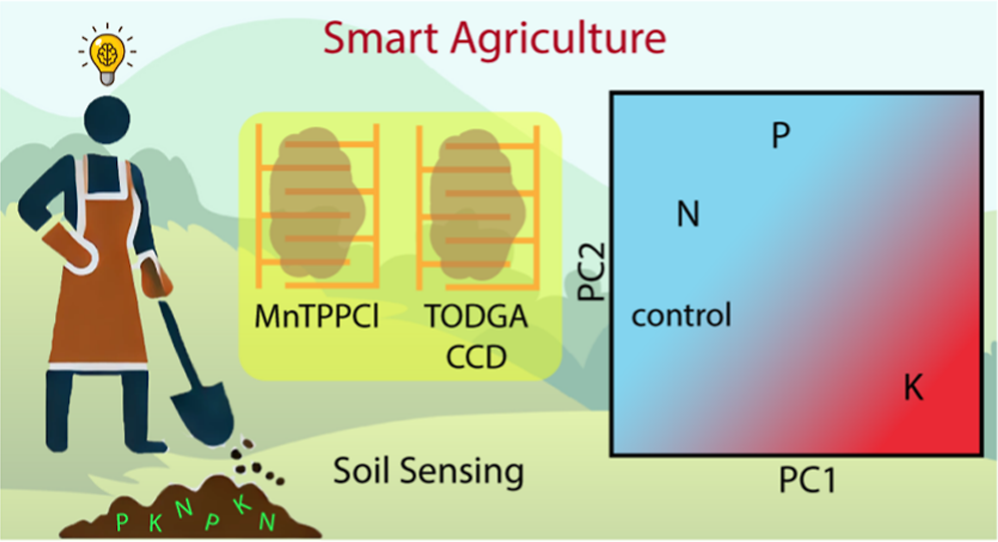

The need to increase food production to address the world population growth can only be fulfilled with precision agriculture strategies to increase crop yield with minimal expansion of the cultivated area. One example is site-specific fertilization based on accurate monitoring of soil nutrient levels, which can be made more cost-effective using sensors. This study developed an impedimetric multisensor array using ion-selective membranes to analyze soil samples enriched with macronutrients (N, P, and K), which is compared with another array based on layer-by-layer films. The results obtained from both devices are analyzed with multidimensional projection techniques and machine learning methods, where a decision tree model algorithm chooses the calibrations (best frequencies and sensors). The multicalibration space method indicates that both devices effectively distinguished all soil samples tested, with the ion-selective membrane setup presenting a higher sensitivity to K content. These findings pave the way for more environmentally friendly and efficient agricultural practices, facilitating the mapping of cropping areas for precise fertilizer application and optimized crop yield.

## Introduction

The projected global population of 9.8 billion by 2050 necessitates a 70% increase in food production.^1^ To achieve this sustainably, innovative strategies are required to enhance agricultural yield without expanding arable land.^2,3^ Precision agriculture, a key aspect of smart agriculture, emerges as a crucial technique that enables increased productivity through the site-specific application of fertilizers, guided by precise soil nutrient monitoring.^4,5^ Traditionally, soil fertility is assessed by collecting multiple samples from fields for chemical analysis in laboratories, a process that can be expensive and often leads to insufficient sampling, thus failing to accurately represent the spatial variability of soil nutrients.^6^ Recent advancements in soil sensing technologies underscore a shift toward noninvasive and real-time monitoring methods, crucial for both precision and smart agriculture.^7−12^

Recent advancements in smart agriculture emphasize the integration of advanced sensor technologies and the use of machine learning algorithms to enhance the precision and efficiency of farming practices.^7,8^ Wearable sensors developed for applications like leaf moisture monitoring employ capacitive measurements to provide real-time data on plant health, demonstrating significant advances in noninvasive agricultural monitoring.^9,10^ The integration of internet of things and artificial intelligence technologies in agriculture not only improves productivity but also contributes to sustainable farming practices by minimizing resource wastage and environmental impact. Moreover, the deployment of multisensor arrays has proven their versatility across various agricultural applications, including the precise detection of soil nutrients and environmental contaminants, boosting a more rational and rapid decision-making action in the future.^11−13^

Multisensor arrays, particularly effective in analyzing complex fluids, transform raw data into recognizable patterns through advanced computational and statistical methods.^14−16^ These arrays have proven their utility in various sectors, including food and beverage quality control,^17−20^ and the detection of pollutants and biomedical markers.^13,21−23^ Specifically, potentiometric and impedimetric multisensor arrays have shown great promise in soil analysis.^24−28^ For instance, an impedimetric multisensor array based on layer-by-layer (LbL) coatings showed good distinction of soil samples enriched with nitrogen (N), phosphorus (P), potassium (K), calcium (Ca), magnesium (Mg) and sulfur (S), all of them easily dispersed in water with raw impedance data processed by distinct multidimensional projection techniques for improved information visualization.^26^ In a similar context, Khaydukova et al. applied a potentiometric multisensor array based on cross-sensitive plasticized polymeric membranes to determine the NPK content in aqueous soil dispersions, with the main advantage of a fast-measuring procedure (only 8 min for data acquisition).^25^ These nutrients, crucial for plant growth, are routinely applied each cropping season.

In this study, we explored the capabilities of novel impedimetric multisensor arrays for distinguishing soil samples enriched with essential macronutrients. Using ion-selective membranes and LbL films, we demonstrated the arrays’ ability to effectively classify these samples through simple water dispersion and advanced statistical techniques. The machine learning analysis, particularly decision tree (DT) models, was employed to enhance the classification accuracy, indicating that both devices effectively distinguished all soil samples tested, with the ion-selective membrane setup presenting a higher sensitivity to K content. The findings indicate the potential for these sensor arrays to contribute significantly to in situ soil analysis, paving the way for more precise and affordable nutrient monitoring in support of precision agriculture.

## Materials and Methods

**Figure 1:**
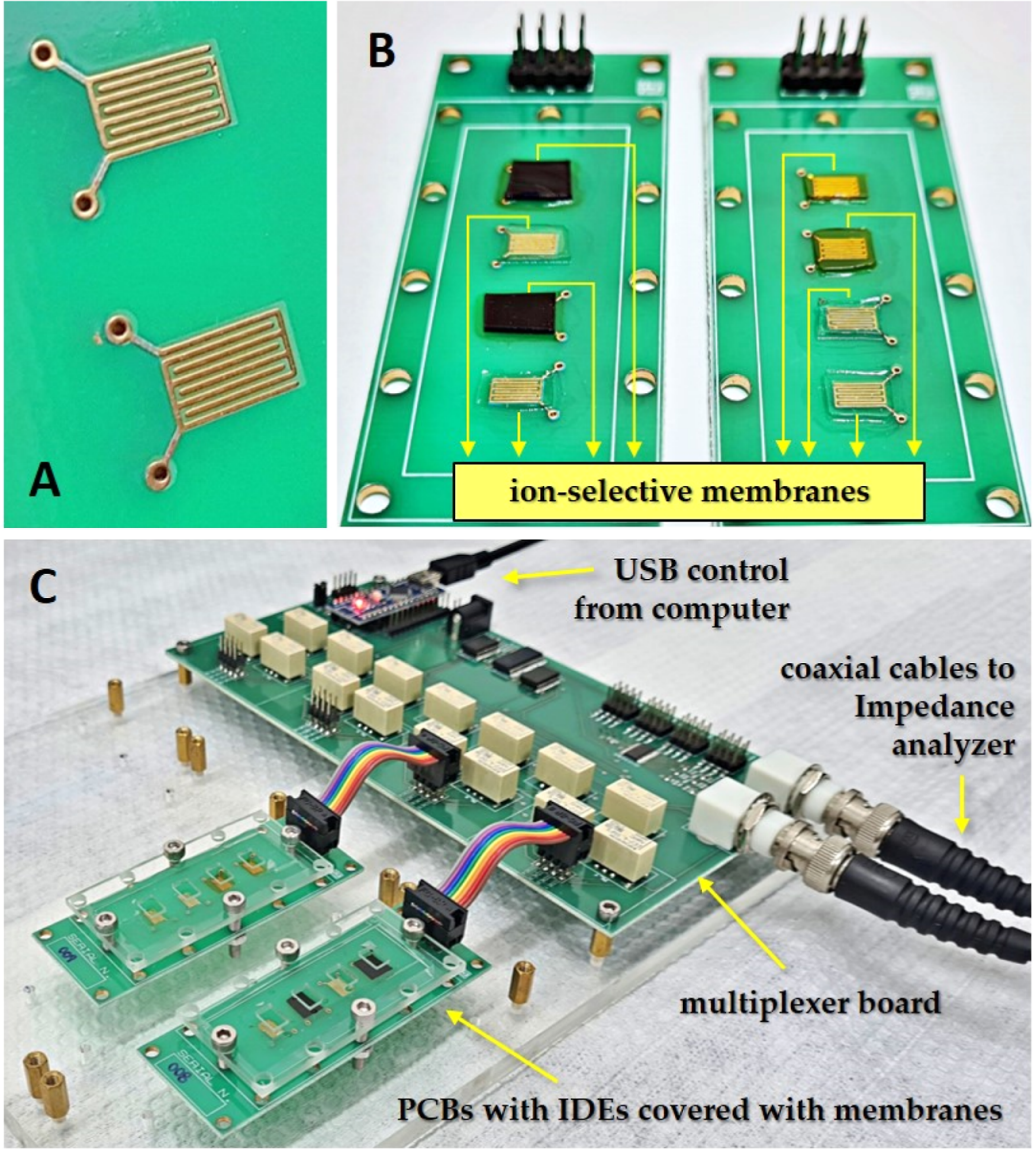
Impedimetric multisensor array: (a) close-up of the bare IDEs before the adhesion of ion-selective membranes. (b) Two PCBs with IDEs covered by eight distinct ion-selective membranes, allowing for simultaneous detection of multiple analytes. (c) The multisensor array setup consists of sensing units connected to a digitally controlled analog multiplexing circuit, enabling precise and efficient impedance measurements. The IDEs have four pairs of digits having 5 mm length, 0.2 mm width, and 0.2 mm spacing.

The impedimetric multisensor array used in this study is not a commercial product, but a custom-built setup. The core of the device is based on four collinear, gold-plated interdigitated electrodes (IDEs), embedded on a printed circuit board, fabricated by TEC–CI Circuitos Impressos (São Paulo-SP, Brazil) according to the authors’ instruction.^29^Figure 1 displays photos of the bare IDEs, the multisensor array, and the setup for impedance measurements. The multisensor array comprises eight sensing units made with ion-selective membranes, five anionic and three cationic membranes, labeled here from S1 to S8, with full description displayed in Table S1 of the Supporting Information. The membranes consist of plasticized polymeric sensor membranes containing poly(vinyl chloride) (PVC) as a polymer, dioctyl sebacate or *o*-nitrophenyloctyl ether (NPOE) as a plasticizer, and various membrane-active compounds listed in Table S1.^30,31^ The membranes were synthesized using a conventional procedure, where the weighed amounts of all components were dissolved in freshly distilled tetrahydrofuran (THF), and the resultant solutions were poured into flat-bottom Teflon beakers to evaporate the solvent. The resulting membranes were cut in ∼(6 × 4) mm^2^ and adhered to the IDEs using THF.

A multisensor array having LbL films^32^ as sensing units is used for comparison, with a picture of the device shown in Figure S1. Full details on the fabrication of this microfluidic device are given elsewhere.^29^ Briefly, copper phthalocyanine-3,4′,4″,4‴-tetrasulfonic acid tetrasodium salt (CuTsPc), montmorillonite clay (MMt-K), poly(3,4-ethylenedioxythiophene)–poly(styrenesulfonate) (PEDOT:PSS), and poly(diallyldimethylammonium chloride) solution (PDDA) were purchased from Sigma-Aldrich and used as received as cationic and anionic layers in LbL assemblies. These materials are simply dispersed in deionized water and the LbL films are fabricated by sequentially alternating their aqueous dispersions onto the IDEs to form 10 bilayers of PDDA/CuTsPc, PDDA/MMt-K, and PDDA/PEDOT:PSS. Three electrodes modified with LbL films and one bare IDE comprise four distinct sensing units for the setup configuration.

Impedance measurements are conducted in a Solartron 1260A impedance/gain-phase analyzer, applying a 25 mV AC voltage over 1–10^6^ Hz frequency range. To facilitate the routing of analog signals between the sensing units and impedance analyzer, a new homemade multiplexer was developed based on previous ones.^29,33^ The software-controlled multiplexer utilized a cascading relay network to enable fully automated and customizable experimental sequences of measurements. In a separate electronic assembly, a digitally controlled analog multiplexing system was designed and built to independently route the signal of up to 16 IDEs (4 PCBs containing 4 IDEs each) to a pair of coaxial BNC connectors. This unit features an inexpensive Arduino Nano microcontroller module, communicating via USB to a PC unit. The Arduino powers an array of small-signal relays through a Darlington driver integrated circuit (ULN2003) from Texas Instruments.

Before employing the sensor arrays for soil analysis, which comprise much more complex samples, we put both in a proof-of-concept test, aiming to distinguish electrolytes and nonelectrolytes solutions representing the five basic tastes relevant to human gustatory perception: sweet, salty, sour, bitter, and umami. To this end, we analyze 1 mM aqueous solutions of sucrose (C_12_H_22_O_11_), sodium chloride (NaCl), hydrochloric acid (HCl), caffeine (C_8_H_10_N_4_O_2_), and l-glutamic acid (C_5_H_9_NO_4_). This molar concentration is close to, or even lower than, some human taste thresholds,^34−37^ with an exception for bitterness that can be perceived at μM concentrations by the biological counterpart.^35^ Both impedimetric multisensor arrays successfully respond to all basic tastes, encouraging us to evaluate their performance in more complex analytes, such as soil samples.

Soil with low natural fertility from Campinas (SP/Brazil), referred here as the “control”, is divided into distinct containers and individually enriched with N, P, and K, using NH_4_NO_3_, NH_4_H_2_PO_4_, and KCl fertilizers, respectively, as described elsewhere.^38,39^ The soil nutrients have been previously analyzed with conventional wet-chemical analysis, with the results presented in Table S2.^40^ Wet-chemical analyses encompass the estimation of K and P availability using the ion-exchange resin extraction method, and total N is estimated through the Kjeldahl method, as previously described.^41^ Note that the soil samples naturally contain N, P and K nutrients in addition to others not mentioned here. Once again, control samples are those not enriched, and named “N”, “P”, and “K” refer to samples enriched with these nutrients, respectively.

The following procedure is adopted for impedance measurements. Initially, control, and N, P, and K enriched samples are dispersed in water at 100 mg/mL. They are sequentially sonicated 30 min in an ultrasound bath, and then left resting for 1 h before analysis. The resulting suspensions are diluted three times to yield four portions of each soil sample at 1, 10, 50, and 100 mg/mL. We need 1 mL for three independent sets of measurements for each sample using the LbL-based array, and 6 mL for triplicates using the ion-selective membranes array. The smaller volume required by the LbL multisensor array is due to the microfluidic integration enabled in that setup. To assess possible cross-contamination before evaluating a new soil sample, the response of a control analyte (distilled water) is evaluated after each soil measurement. The acquisition time for each analyte is ∼5 min for the LbL-based array (4 sensing units) and ∼10 min for the ion-selective membranes array (8 sensing units). The analytes are evaluated at 5 mL/h in the microfluidic device containing LbL films as sensing units, with the flow rate chosen in previous work, with the best discrimination reached without wasting unnecessary amounts of sample.^29^ An example of the time expended in this analysis using the multiplexer, with 8 sensing units based on ion-selective membranes, an analyte was measured in triplicate and with three independent measurements in ∼30 min. Consequently, the entire process for the triplicates in the 16 soil samples studied here (control, N, P and K dispersed in four distinct concentrations in water) can be completed in ∼8 h. This is in stark contrast to individual devices without a multiplexing system, which would require more than a single working day to accomplish the same task with the same soil samples.^26,28^

The impedance raw data presents high dimensionality (*n* ≥ 124) for the multisensor arrays, so we underwent dimensionality reduction using Principal Component Analysis (PCA),^42^ which involved projecting the multidimensional data into a 2D Euclidean space for easier interpretation and visualization. To assess the quality of the resulting data discrimination, we applied the silhouette coefficient (SC)^44^ computed as a value s(*i*) for each object *i* that measures its similarity to its cluster (cohesion), when compared to other clusters (separation). We also calculated the SC^44^ for the entire plot, which is the average of s(*i*) for all objects in the data set. Positive values of SC are interpreted as strong (0.71–1.0), reasonable (0.51–0.70), weak (0.26–0.50), and no discrimination (≤0.25).^44^ Based on the SC scores, we used the *k*-means algorithm^45^ to identify the number (*k*) of nonoverlapping clusters in an unbiased manner. PCA and SC scores are complementary approaches in data analysis, with the first being a dimensionality reduction technique largely used to identify patterns and structures in the data, while SC provides a value indicating how well an object is allocated in a cluster (it assesses the quality of clustering). The raw impedance data are processed using the Orange Data Mining software to perform PCA, compute SC scores, and execute *k*-means clustering.^46^

Classification models from machine learning methods combined with visual representations are employed for analyzing the data from the multisensor arrays. For each multisensor array a multidimensional calibration space (MCS)^16^ is produced by taking the impedance magnitude value (Ohms) in different frequencies. Thus, for distinguishing soil samples categorized into 4 classes (control, K, N, and P), one MCS is created for the multisensor array made with LbL films, and another for the ion-selective membrane-based array. DT models^47−49^ are used for creating such calibrations, using ExMatrix for interpretability.^50^ A model selection experiment is conducted via KFold Cross-Validation^51^ to choose the hyperparameter combinations that maximize DT models’ performance. Then, the hyperparameters holding the highest average performance from the KFold Cross-Validation are applied to create the final DT model using all data. To obtain the deployed (final) DT model’s average performance, a Nested KFold Cross-Validation is conducted.^52,53^ In this procedure, two KFold Cross-Validation loops (inner and outer) are executed. The inner loop assesses model hyperparameter tuning (model selection),^48,49^ whereas the outer loop involves model performance estimation.^52,53^ Optimistic (overestimation) and biased performance measures are typical issues on small data sets, and they can be mitigated using a Nested KFold Cross-Validation.^52,53^ The MCS provides interpretability, unveiling associations among the impedance magnitude values and classes (e.g., soil samples category), and the Nested KFold Cross-Validation method delivers a more truthful performance estimation.^43^

## Results and Discussion

**Figure 2:**
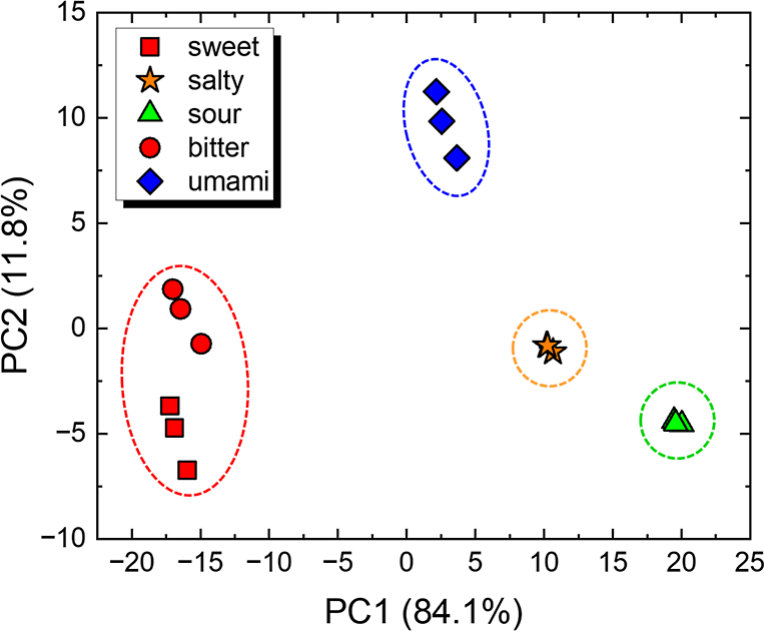
PCA score plots from impedance data in three independent sets of measurements evaluating basic tastes relevant to human gustative perception (sweet, salty, sour, bitter, and umami) at 1 mM obtained with the multisensor array based on ion-selective membranes.

Both multisensor arrays can easily distinguish solutions responsible for relevant basic tastes (sweet, salty, sour, bitter, and umami) by processing the impedance raw data in PCA score plots. Some examples of the impedance spectra obtained are shown in Figure S2. Figure 2 shows the PCA plot for the multisensor array made with ion-selective membranes, while the PCA plot corresponding to the LbL-based array is shown in Figure S3 in the Supporting Information The sum of the explained variance for the first two principal components yielded 97.0 and 95.9% of the total information for the data sets obtained with the devices made with LbL films and ion-selective membranes, respectively. The different shapes in the markers of the scatter plot correspond to the sort of sample, while the different colors indicate the clustering suggested by the *k*-means method considering the highest SC score. Even though one can visually distinguish all five taste samples with both setups, the *k*-means clustering indicates four groups maximizing the discrimination (SC = 0.789) for the ion-selective membrane-based device, with the algorithm combining sweetness (sucrose) and bitterness (caffeine) in a single cluster (red area). As the membranes are ion-selective, it is anticipated that they perform well with electrolytes but not as effectively with nonelectrolytes. This expectation is confirmed by these results, where the PCA clusters caffeine and sucrose together, while distinctly separating the other tastes. In contrast, due to their nonion-specific nature, the LbL-based array demonstrates a superior ability to distinguish between all five taste samples, including sucrose and caffeine, reflected in higher SC scores and distinct clustering in the PCA plots (Figure S3). When five clusters are considered, the SC values are 0.823 and 0.722 for the arrays based on LbL films and ion-selective membranes, respectively. Despite the decrease in the SC score from 0.789 to 0.722 when five clusters are considered for the ion-selective membrane-based data set, this score remains above 0.7, indicating robust discrimination within the clusters.^44^

**Figure 3:**
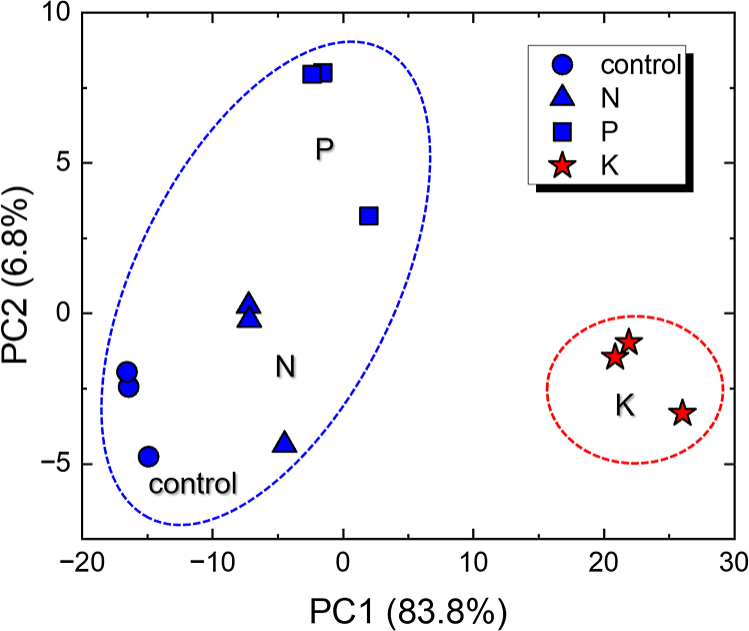
PCA score plots of the impedance data in three independent sets of measurements to evaluate soil samples individually enriched with N, P, and K. Data sets obtained from the evaluation of the soil samples dispersed in water at 100 mg/mL with the multisensor array made with ion-selective membranes. The PCA score plots for soil samples at 1, 10, and 50 mg/mL presented a similar pattern and are shown in the Supporting Information (Figure S4).

The analysis of impedance data for soil samples facilitated the distinction between control, N, P, and K samples across all tested concentrations (1, 10, 50, and 100 mg/mL), as depicted in the PCA score plots. Figure 3 illustrates the PCA score plot for the 100 mg/mL data set obtained with the ion-selective membrane array, with additional plots for the other concentrations and the LbL-based array presented in Figures S4 and S5, respectively. The PCA score plots for the ion-selective membrane array enabled a visual distinction between the control sample and those enriched with N, P, and K in Figure 3. However, *k*-means clustering suggests that maximal discrimination is achieved in two clusters: one combining the control with N and P samples (blue area), and another exclusively containing the K-enriched sample (red area). The results indicate that the ion-selective membranes exhibit particular sensitivity to potassium, likely due to its prevalent ionic state which enhances its mobility and solubility, thereby facilitating detection at the electrode/electrolyte interface. This sensitivity could be instrumental for the selective detection of potassium in soil samples, offering a significant step toward the precise quantification of this essential macronutrient.

In contrast, the PCA plots for the LbL-based device (Figure S5) show that all soil samples are visually distinguishable across aliquots of 1, 10, 50, and 100 mg/mL. This is expected, as the LbL-based array, unlike the ion-selective membrane array, does not employ materials that are selectively sensitive to specific ions. Specifically, for the 1 mg/mL aliquot, *k*-means clustering and SC scores reveal that the control and P samples cluster together, while N and K form a separate cluster. This pattern, previously observed by Americo da Silva et al. using an LbL-based sensor array, underscores the differential sensitivity to N and K over P.^28^ Due to the straightforward sample preparation method, involving only the dispersion of soil in water without any chemical pretreatments, phosphorus tends to remain bound within soil colloids as complex compounds. This results in its poor solubility and minimal contribution to the differentiation in PCA plots, which explains the proximity of P-enriched samples to the control.

The method of simple dispersion in water, while aiding rapid and noncomplex field measurements, does not fully address the detection of nitrogen and phosphorus in their varied and complex forms within the soil. Future improvements should incorporate more sophisticated extraction techniques to enhance detection capabilities for nitrogen and phosphorus, as well as other nutrients, recognizing that the current approach may limit the accuracy and sensitivity particularly for nitrogen, given its presence in hydrolyzed and insoluble forms. The forthcoming enhancements to the extraction methods will be crucial to optimize the sensor arrays’ performance and to ensure more reliable, sensitive measurements of soil nutrients.

Table 1 presents the results with the two multisensor arrays for the control, N, P, and K soil samples dispersed in water at 1, 10, 50, 100 mg/mL, including the number of clusters suggested by the *k*-means method, the SC values used to find the best clustering in each case, and the contributions from the first (PC1) and second (PC2) principal components. Regardless of the soil sample dilution rate, most information is concentrated on PC1, above 66% for all data sets and higher for the data obtained with the multisensor array made with LbL films. We observed a trend in the data sets from the ion-selective membrane array, where the contribution of PC1 increased in proportion to the concentration of the soil aliquots, as detailed in Table 1. However, caution is warranted, as more than one physical or chemical variable may be influencing a principal component.

**Table 1:** Number of Clusters Found With the *k*-Means Method, SC Scores, and Contributions from PC1 and PC2 in the Impedance Data for Soil Samples Dispersed in Water at 1, 10, 50, and 100 mg/mL, Obtained with the LbL-Based and Ion-Selective Membrane-Based Arrays

	LbL films				ion-selective membranes			
data sets (mg/mL)	1	10	50	100	1	10	50	100
number of clusters (*k*)	2	4	4	4	2	2	2	2
silhouette coefficient	0.585	0.694	0.810	0.841	0.401	0.505	0.634	0.624
PC1 (%)	72.3	88.5	88.0	85.7	66.1	79.6	83.8	83.8
PC2 (%)	14.7	7.3	8.0	7.8	13.0	11.2	7.9	6.8
PC1 + PC2 (%)	87.0	95.8	96.0	93.5	79.1	90.8	91.7	90.6

**Figure 4:**
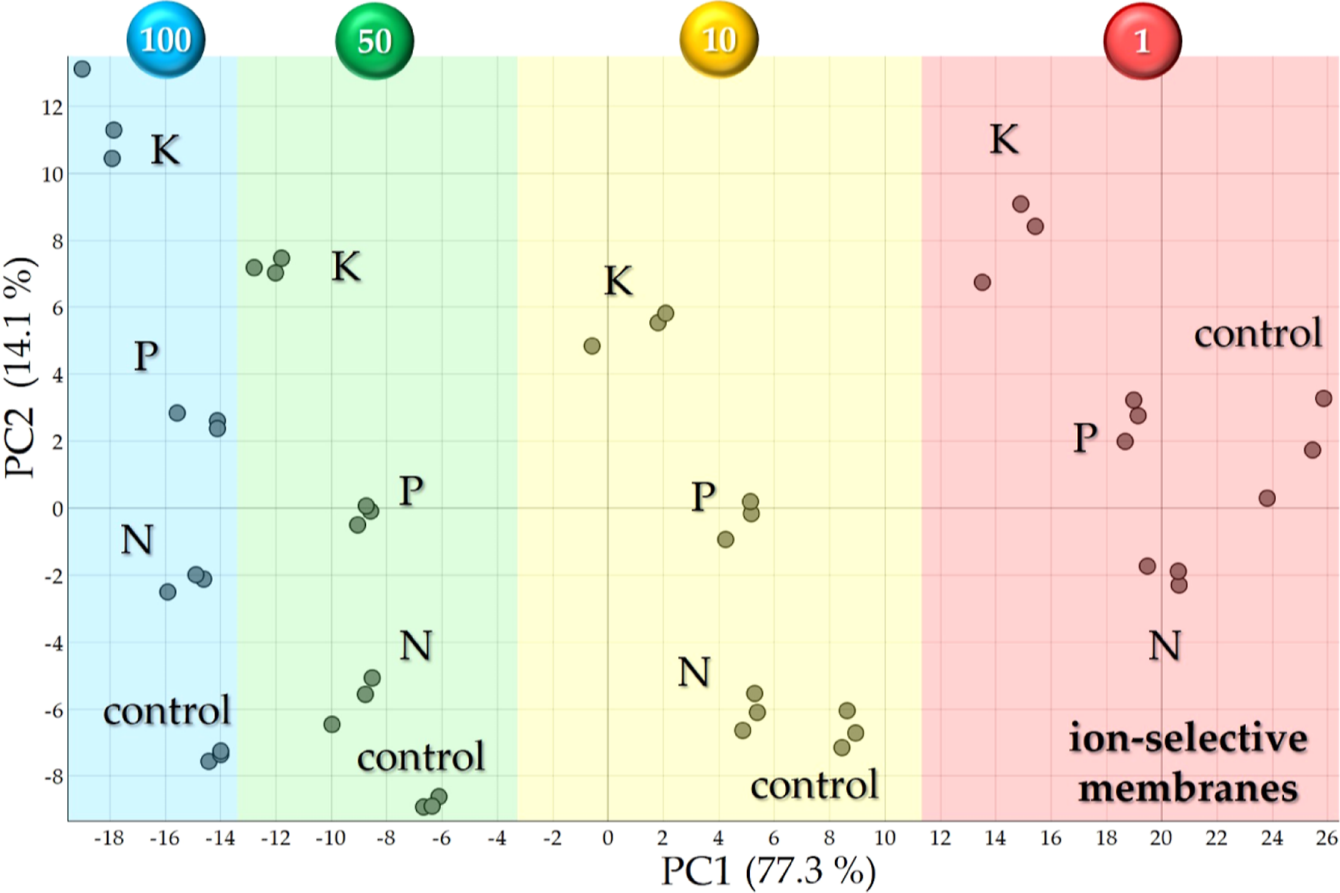
PCA score plots of the impedance data of control, N, P, and K soil samples dispersed in water at 1 (red), 10 (yellow), 50 (green), and 100 (blue) mg/mL obtained with the multisensor array made with ion-selective membranes.

Figure 4 displays all data sets obtained with the multisensor array made with ion-selective membranes for the soil samples at four concentrations, featuring a concentration trend in PC1 with increasing concentrations positioned from right to left. Red, yellow, green, and blue areas in the graphs are related to soil samples prepared at 1, 10, 50, and 100 mg/mL, respectively. A similar trend was observed by Americo da Silva et al. for sandy and clayey soils, with PCA plots indicating a PC1 trend related to soil fertility.^28^ As clearly seen in Figure 4 it is easy to discriminate all samples from the ion-selective membrane array, while the visual analysis of the data from the LbL-based array is not straightforward as a strong contribution from PC2 results in a boomerang-like pattern (Figure S6). The boomerang-shaped pattern observed in Figure S6 demonstrates a nonlinear response in the PC1 vs. PC2 plot across different concentrations of soil samples dispersed in water. This distinct pattern indicates that the LbL-based array achieves optimal clustering performance at certain concentration thresholds, thereby optimizing the detection efficacy for this specific set of soil samples and sensor configuration. For instance, the LbL-array fails to distinguish all soil macronutrients at 1 mg/mL using the *k*-means clustering method (Figure S5a). Additionally, the sample enriched with K at 50 mg/mL clusters in the same region as the 100 mg/mL samples (Figure S6). These results emphasize that the 10 mg/mL concentration offers optimal clustering performance for the LbL-array.

One of the most relevant challenges in sensor arrays is calibration, which can be troublesome when one of the sensing units of the array must be replaced. This can be partially addressed by using the concept of MCS^16^ which is based on machine learning methods rules, providing prediction power along with interpretability (i.e., explainability). In addition to assigning the class (e.g., soil samples category) in a forecasting task, these calibrations allow for establishing relationships between the classes and the impedance values and specific sensing units. For distinguishing soil samples into 4 classes (control, N, P, and K), two MCS are created from the impedance modulus spectra, one for the LbL-based device and the other for the ion-selective membranes setup. Each sensing unit provides the impedance magnitude at 31 frequencies (F1 to F10^6^ Hz), resulting in 124 frequencies for the LbL-based array comprising 4 sensing units (bare IDE, PDDA/CuTsPc, PDDA/MMt-K, and PDDA/PEDOT:PSS), and 248 frequencies for the ion-selective membrane array that integrates 8 sensing units (named S1 to S8, whose compositions are given in Table S1 of the Supporting Information). The calibrations (MCS) are generated by DT models for classifying samples among control, N, P, and K (target variable - soil categories) classes,^48,49^ with models’ hyperparameters and accuracy (performance) obtained via the Nested KFold Cross-Validation procedure.^52,53^ The estimated average accuracy—i.e., model’s performance in classifying samples among classes (control, N, P, and K)—is 91.5% for the ion-selective membrane array, while a 87% accuracy is achieved for the LbL-based array. These averages are not statistically different (*p*-value of 0.52578); therefore, based on accuracy both devices perform similarly.

**Figure 5:**
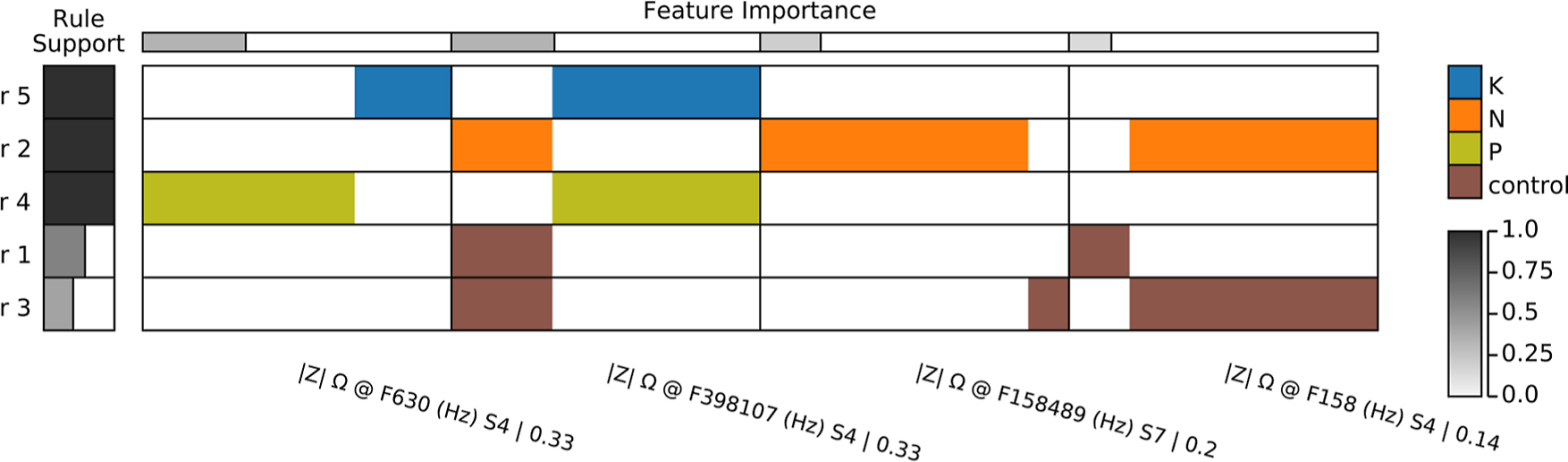
MCS for the multisensor array based on ion-selective membranes. The space has 4 dimensions corresponding to 4 frequencies (features) selected among the 248 available (F1 to F10^6^ Hz for 8 sensors). Rules with maximum support are found, e.g. rules r5, r2, and r4 (first to the third row), distinguishing all samples from classes K (blue), N (orange), and P (olive), respectively. Only two sensors in the multisensor array are used, namely sensor S4 at the frequencies F630, F398107, and F158 (first, second, and fourth columns) and sensor S7 at frequency F158489 (third column). The average accuracy estimated for the MCS via the DT model is 91.5%.

The MCS can be visually represented and interpreted using the ExMatrix approach.^50^ In this representation, logic rules derived from DT models are displayed in a matrix-like visual metaphor, where rows represent rules, columns signify features, and cells indicate rule predicates. These cells define the range of impedance magnitude values for different classes, such as soil sample categories, which are displayed using color coding. Figure 5 illustrates the ExMatrix representation for the MCS developed using the ion-selective membrane-based multisensor array, with the corresponding MCS for the LbL films array shown in Figure S7. Within the ExMatrix, rules (rows) are organized by class and support level, while features (columns) are ranked by importance. The MCS for the ion-selective membrane array includes four selected frequencies from the 248 available, spanning from F1 to F10^6^ Hz across eight sensors. Notably, data from only two sensing units, S4 and S7, are utilized among the eight available. The ion-selective capturing mechanisms of these membranes leverage specific ion-exchange interactions, where active compounds within the membranes bind selectively to target ions in the sample. Frequencies F630, F398107, and F158 emanate from the S4 sensing unit, which incorporates Mn(III) tetraphenylporphyrin chloride (MnTPPCl) for anion exchange, particularly effective with chloride ions from KCl, and tetradodecylammonium bromide that adjusts membrane surface properties affecting ion transport. The frequency F158489 originates from the S7 sensing unit, employing tetraoctyl diglycolamide (TODGA) for its strong cation-exchange capacity with potassium ions, enhanced by chlorinated cobalt dicarbollide, which further aids in selectively capturing potassium. This dual-component strategy in S7 enhances its sensitivity and selectivity for potassium, showcasing the intricate balance of membrane composition and its impact on device performance.

For the LbL-based array, the MCS has 13 dimensions, i.e. thirteen selected frequencies among the 124 (F1 to F10^6^ Hz for 4 sensing units). At least two frequencies (feature/column) from each sensing unit (bare IDE, PDDA/CuTsPc, PDDA/MMt-K, and PDDA/PEDOT:PSS) are used. The rules in the MCS for ion-selective membranes are more generic (higher support) than the rules for the LbL-based array. Indeed, rules r5, r2, and r4 (first to the third row in Figure 5) have maximum support, i.e. they permit distinction of all soil samples from classes K (blue), N (orange), and P (olive), respectively. Ranges of impedance values from such rules can be interpreted regarding class associations. Taking rule r5 (first row), for example, class K (blue) is associated with soil samples containing high impedance (right-most rectangle) at the F630 frequency (first column), and medium to high values (center-to-right rectangle) at the F398107 frequency (second column), both for sensing unit S4. No rules from the MCS obtained with the LbL array have maximum support and that explains a certain data complexity to acquire rules distinguishing samples among their 4 possible classes.

In a nutshell, the calibrations obtained for the two multisensor arrays are typical examples of the potential of the MCS approach, relying on interpreting the machine learning model instead of only inspecting quantitative measurements. Both calibrations have similar performance (e.g., accuracy), but the MCS for the ion-selective membrane-based array (Figure 5) is simpler (4 dimensions) and more generic (rules with high support) than the MCS for the LbL-based device (13 dimensions and not having high support rules—Figure S7). Based on the calibrations created, the data (impedance at different frequencies) produced by the ion-selective membrane-based array present a better fingerprint of the soil samples among 4 classes/categories (control, K, N, and P). It is worth mentioning that frequencies and sensors employed in both calibrations (Figures 5 and S7) are chosen by DT model inference algorithm. Nevertheless, it is not possible to state unambiguously that other frequencies and sensor selections could not produce equivalent models’ performance.

## Conclusions

In conclusion, this study introduces a novel impedimetric multisensor array featuring ion-selective membranes that successfully discriminates between soil samples enriched with various macronutrients. The simplicity of the sample preparation—i.e., mere dispersion in water—eliminates the need for complex preprocessing, enhancing the practicality of soil analysis. The distinct profiles of these samples are evident from visual PCA and further supported by comparative results from another array utilizing LbL films. Utilization of *k*-means clustering on the impedance data reveals that although the LbL film-based array can statistically cluster and discriminate all samples, the ion-selective multisensor array exhibits exceptional sensitivity to K-enriched samples, clearly differentiating it from samples enriched with N and phosphorus P.

The calibration results from both sensor arrays highlight the versatility of the MCS, which prioritizes the interpretation of machine learning models instead of solely inspecting quantitative measurements. Although both arrays show comparable performance metrics, such as accuracy, the MCS for the ion-selective membrane-based array is notably simpler and more universal than that for the LbL film-based array. Specifically, the ion-selective sensor array, utilizing data from sensors S4 and S7—which are designed to allow ion exchange with K^+^ and/or Cl^–^—provides a more definitive fingerprint of the soil samples across four categories (control, N, P, and K). These findings align with the observations from both PCA and *k*-means analysis.

While this array does not yet enable specific nutrient recognition, its heightened sensitivity toward K-enriched samples is a significant step toward selective detection, which is essential for identifying individual macronutrients in soil. These promising results open avenues for further research into developing new multisensor arrays for in situ soil analysis. Such technology promises a quick, economical method for supporting precision agriculture by simply diluting soil samples in water.

## Supporting Information Available

Table S1: Composition of the sensor array based on ion-selective membranes. Table S2: Soil composition regarding nitrogen, phosphorus, and potassium availability, according to wet-chemical analysis. Figure S1: Multisensor array based on LbL films and microfluidic device. Figure S2: Representative examples of impedance magnitude spectra obtained for the multisensor array based on 8 ion-selective membranes for NaCl and sucrose analytes. Figure S3: PCA score plots from impedance data in three independent sets of measurements evaluating basic tastes relevant to human gustative perception (sweet, salty, sour, bitter, and umami) at 1 mM obtained with the multisensory array based on LbL films. Figure S4: PCA score plots of the impedance data in three independent sets of measurements to evaluate soil samples individually enriched with N, P, and K. The data sets are obtained from the evaluation of the soil samples dispersed in water at (A) 1, (B) 10, (C) 50 and (D) 100 mg/mL with the multisensory array based on ion-selective membranes. Figure S5: PCA score plots of the impedance data in three independent sets of measurements to evaluate soil samples individually enriched with N, P, and K. The data sets are obtained from the evaluation of the soil samples dispersed in water at (A) 1, (B) 10, (C) 50 and (D) 100 mg/mL with the multisensory array based on LbL films. Figure S6: PCA score plots of the impedance data of control, N, P, and K soil samples dispersed in water at 1 (red), 10 (yellow), 50 (green), and 100 (blue) mg/mL obtained with the multisensory array based on LbL films. Colored areas are just a guide to the eye, once soil sample enriched with K at 50 mg/mL is in the blue area merged with the aliquots at 100 mg/mL (highlighted in the green ellipse). Figure S7: MCS for the multisensor array based on LbL films. The space has 13 dimensions corresponding to 13 frequencies (features) selected among the 124 available (F1 to F10^6^ Hz for 4 sensors). Rules with maximum support are not found, which may indicate a certain data complexity, and at least two frequencies from each sensor employed in the multisensor array (bare IDE, PDDA/CuTsPc, PDDA/MMt-K, and PDDA/PEDOT:PSS) are used. The average accuracy estimated for the MCS via the DT model is 87% (PDF)

## List of Abbreviations and Symbols

AC: alternating current
Ca: calcium
CuTsPc: copper phthalocyanine-3,4′,4″,4‴-tetrasulfonic acid tetrasodium salt
DC: direct current
DOS: dioctyl sebacate
DT: decision trees
HCl: hydrochloric acid
IDE: interdigitated electrode
IDMAP: interactive document mapping
K: potassium
LbL: layer-by-layer
MCS: multidimensional calibration space
Mg: magnesium
MMt-K: montmorillonite clay
N: nitrogen
NaCl: sodium chloride
NPOE: *o*-nitrophenyloctyl ether
P: phosphorus
PCA: principal component analysis
PCB: printed circuit board
PC1: first principal component
PC2: second principal component
PDDA: poly(diallyldimethylammonium chloride) solution
PDDA/CuTsPc: layer-by-layer film of PDDA and CuTsPc
PDDA/MMt-K: layer-by-layer film of PDDA and MMt-K
PDDA/PEDOT:PSS: layer-by-layer film of PDDA and PEDOT:PSS
PEDOT:PSS: poly(3,4-ethylenedioxythiophene)–poly(styrenesulfonate)
PVC: poly(vinyl chloride)
r1 to r15: logic rules represented from 1 to 15 rows in a matrix
S: sulfur
SC: silhouette coefficient
s(*i*): silhouette value
S1 to S8: ion-selective membranes from 1 to 8, according to compounds presented in Table S1
THF: tetrahydrofuran
