# Supplementary material for: Analysis of Macronutrients in Soil Using Impedimetric Multisensor Arrays
Source: ACS Omega. 2024 Jul 25;9(31):33949–58. doi: 10.1021/acsomega.4c04452 (PMC11307303; doi:10.1021/acsomega.4c04452)
Supplement: Supplementary file 1 — ao4c04452_si_001.pdf [file ao4c04452_si_001.pdf]

Supporting Information

Analysis of Macronutrients in Soil Using Impedimetric Multisensor Arrays

Maria Luisa Braunger[a], Mario Popolin Neto[b], Dmitry Kirsanov[c,d], Igor Fier[e], Lucas R. Amaral[f], Flavio M. Shimizu[a], Daniel S. Correa[g], Fernando V. Paulovich[h], Andrey Legin[c,d], Osvaldo N. Oliveira Jr.[i], Antonio Riul Jr.[a*]

[a] Instituto de Física "Gleb Wataghin" (IFGW), Universidade Estadual de Campinas – UNICAMP, 13083-859, Campinas, SP, Brazil.

[b] Federal Institute of São Paulo – IFSP, 14804-296, Araraquara, SP, Brazil.

[c] Institute of Chemistry, St. Petersburg State University, Mendeleev Center, Universitetskaya nab.7/9, 199034 St. Petersburg, Russia.

[d] Laboratory of Artificial Sensory Systems, ITMO University, Kronverkskiy pr, 49, 197101 St. Petersburg, Russia.

[e] Quantum Design Latin America, 13080-655, Campinas, SP, Brazil.

[f] School of Agricultural Engineering (FEAGRI), University of Campinas - UNICAMP, 13083-875, Campinas, SP, Brazil.

[g] Nanotechnology National Laboratory for Agriculture (LNNA), Embrapa Instrumentação, 13560-970, São Carlos, SP, Brazil

[h] Department of Mathematics and Computer Science, Eindhoven University of Technology (TU/e), 5600 MB Eindhoven, The Netherlands.

[i] São Carlos Institute of Physics (IFSC), University of São Paulo - USP, 13566-590, São Carlos, SP, Brazil.

* Corresponding author: riul@unicamp.br

Table S1: Composition of the sensor array based on ion-selective membranes.

Sensing unit code	Membrane active compounds	Plasticizer*	Reference
**S1**	Carbonate ionophore I, tetradodecylammonium bromide	NPOE	[1,2]
**S2**	Sulfate ionophore I, tridodecylmethylammonium nitrate	NPOE	[1,3,4]
**S3**	Mn(III) tetraphenylporphyrine chloride	NPOE	[1,5]
**S4**	Mn(III) tetraphenylporphyrine chloride, tetradodecylammonium bromide	NPOE	[1,5]
**S5**	Hydrosulfite ionophore I, tridodecylmethylammonium nitrate	NPOE	[1,3,6]
**S6**	Potassium tetrakis[3,5-bis(trifluoromethyl)phenyl]borate	DOS	[7]
**S7**	Tetraoctyl diglycolamide, chlorinated cobalt dicarbollide	NPOE	[8]
**S8**	Carbamoyl methylen phosphine oxide, chlorinated cobalt dicarbollide	NPOE	[8]

*The membranes consist of plasticized polymeric sensor membranes containing poly(vinylchloride) (PVC) as a polymer, dioctyl sebacate (DOS) or o-nitrophenyloctyl ether (NPOE) as a plasticizer, and various membrane-active compounds listed in this table.

Table S2: Soil composition regarding nitrogen, phosphorous, and potassium availability, according to wet-chemical analysis.

Soil sample	Nitrogen (mg kg^{-1})	Phosphorous (mg dm^{-3})	Potassium ($mmol_c$ dm^{-3})
**control**	1386	6	1
**N**	**1540**	6	1
**P**	NA*	**88**	1.2
**K**	NA*	5	**6.8**

*The analysis of nitrogen concentration was omitted for the soil samples labeled "P" and "K," as they lacked a direct nitrogen fertilizer source. Therefore, the nitrogen content in these samples is expected to closely resemble that of the "control" sample.

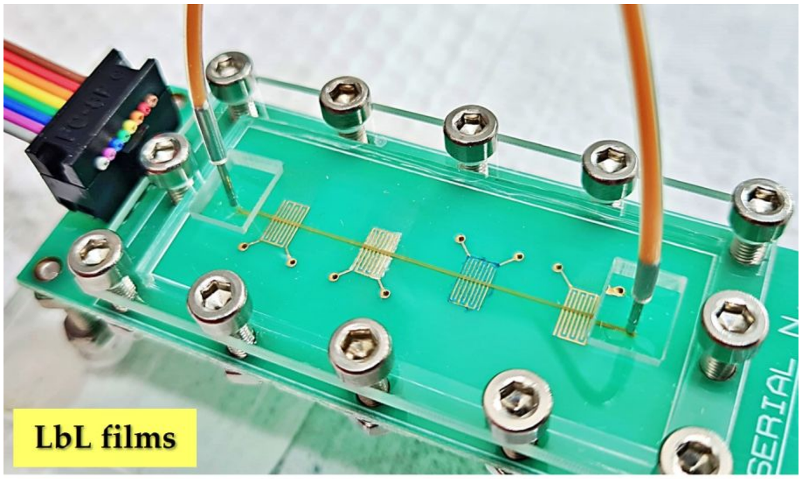

Figure S1: Multisensor array based on LbL films and microfluidic device.

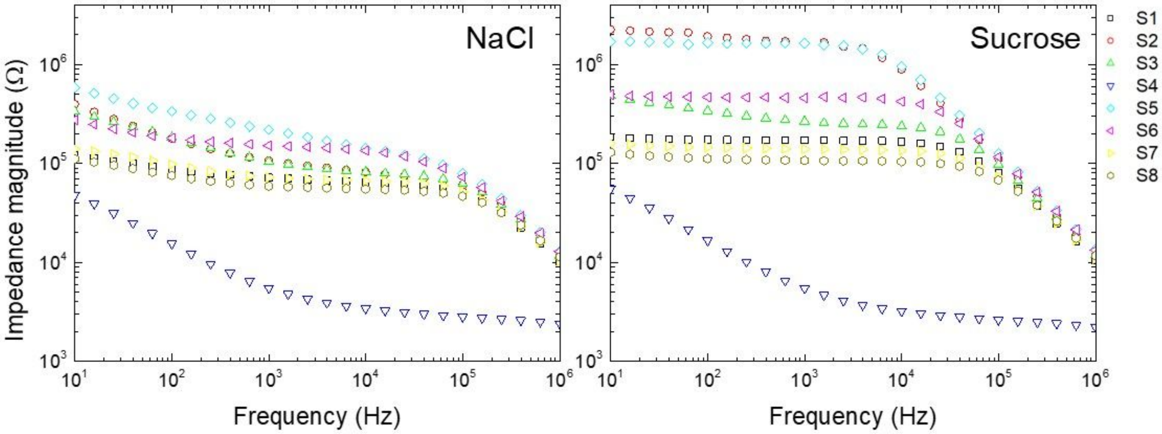

Figure S2: Representative examples of impedance magnitude spectra obtained for the multisensor array based on 8 ion-selective membranes for NaCl and sucrose analytes.

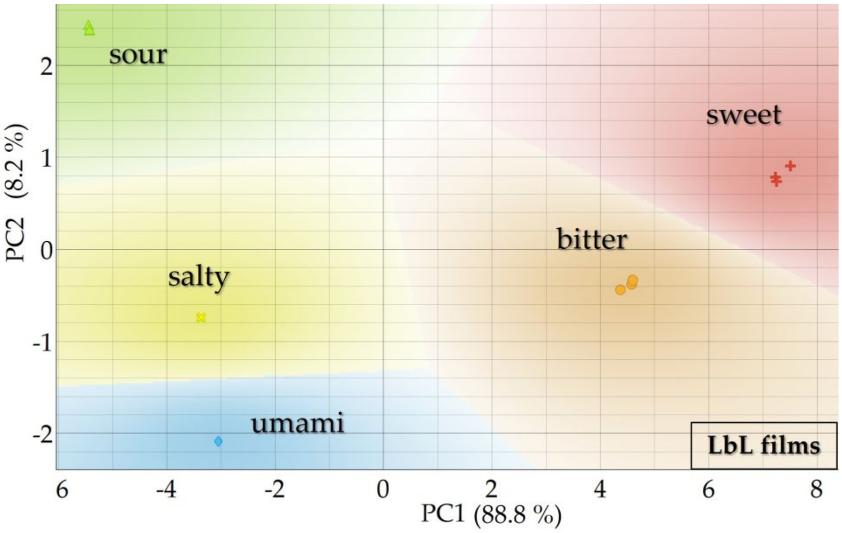

Figure S3: PCA score plots from impedance data in three independent sets of measurements evaluating basic tastes relevant to human gustative perception (sweet, salty, sour, bitter, and umami) at 1 mM obtained with the multisensory array based on LbL films.

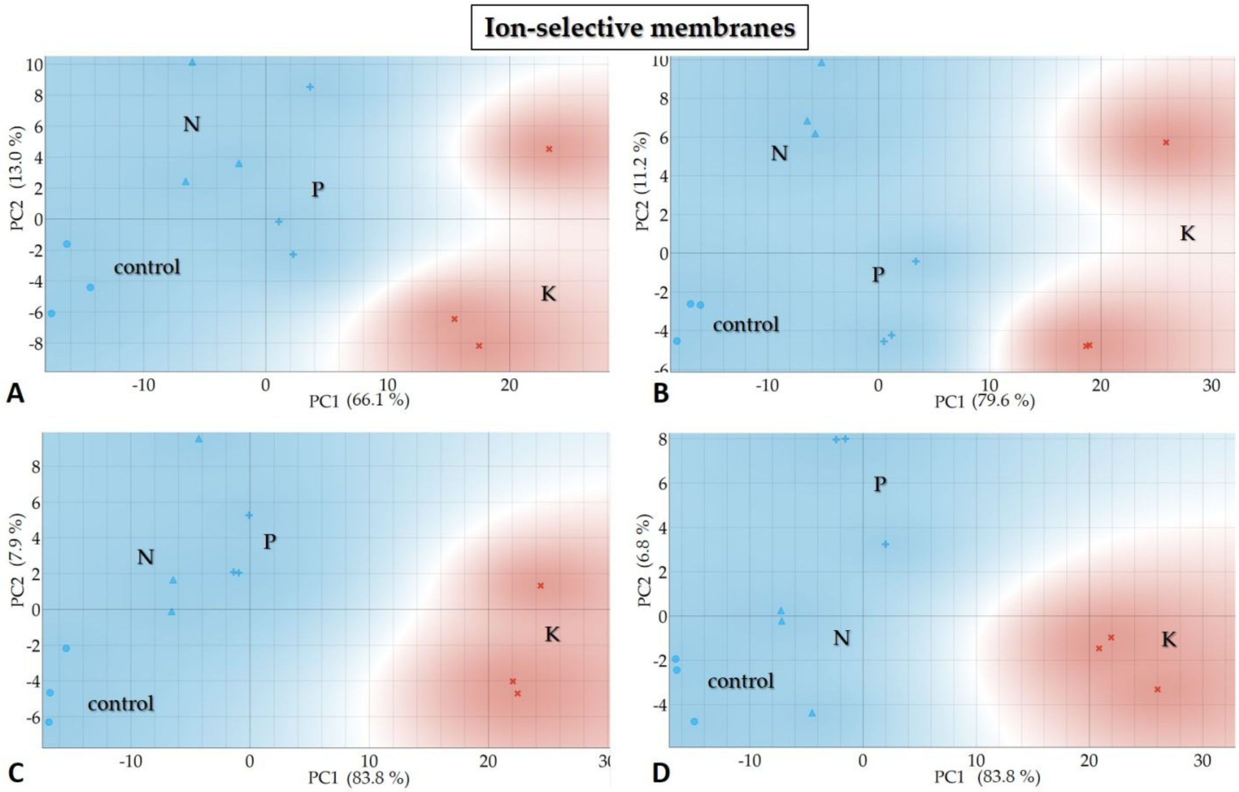

Figure S4: PCA score plots of the impedance data in three independent sets of measurements to evaluate soil samples individually enriched with N, P, and K. The datasets are obtained from the

evaluation of the soil samples dispersed in water at **(A)** 1, **(B)** 10, **(C)** 50 and **(D)** 100 mg/mL with the multisensory array based on ion-selective membranes.

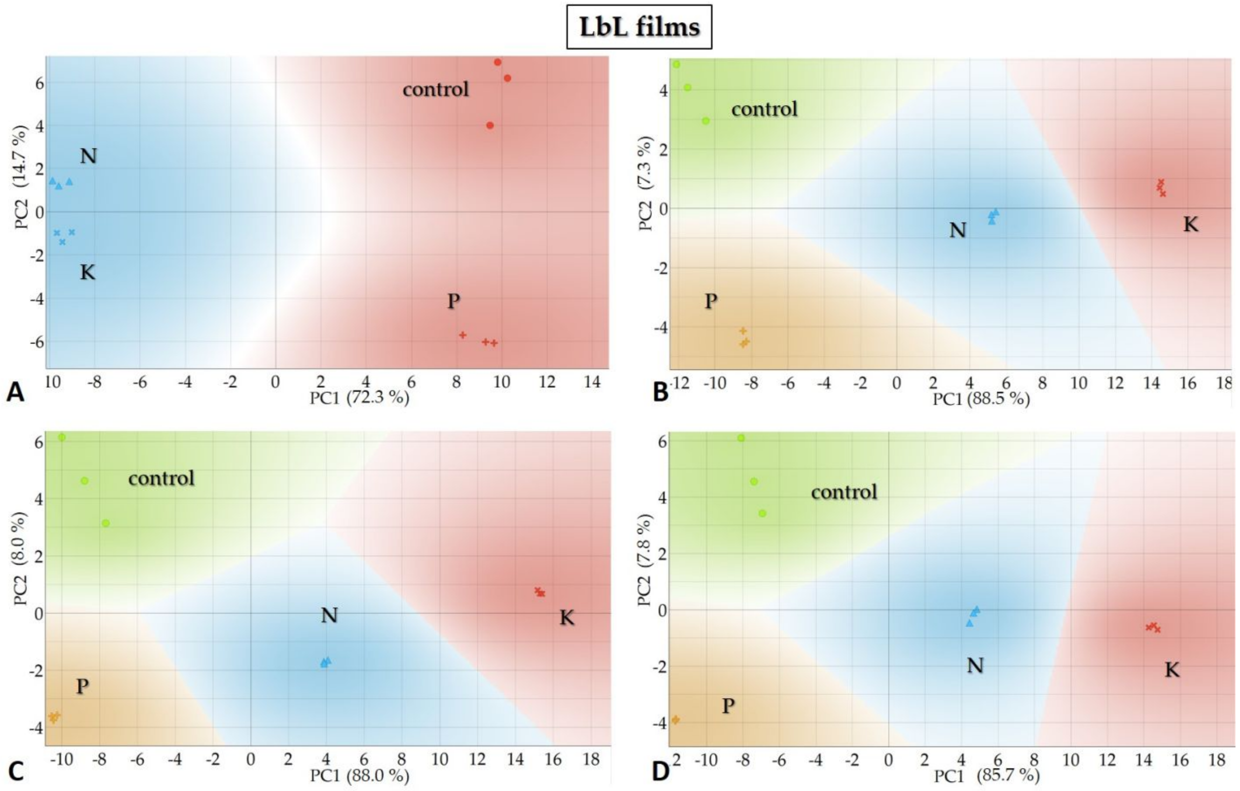

Figure S5: PCA score plots of the impedance data in three independent sets of measurements to evaluate soil samples individually enriched with N, P, and K. The datasets are obtained from the evaluation of the soil samples dispersed in water at **(A)** 1, **(B)** 10, **(C)** 50 and **(D)** 100 mg/mL with the multisensory array based on LbL films.

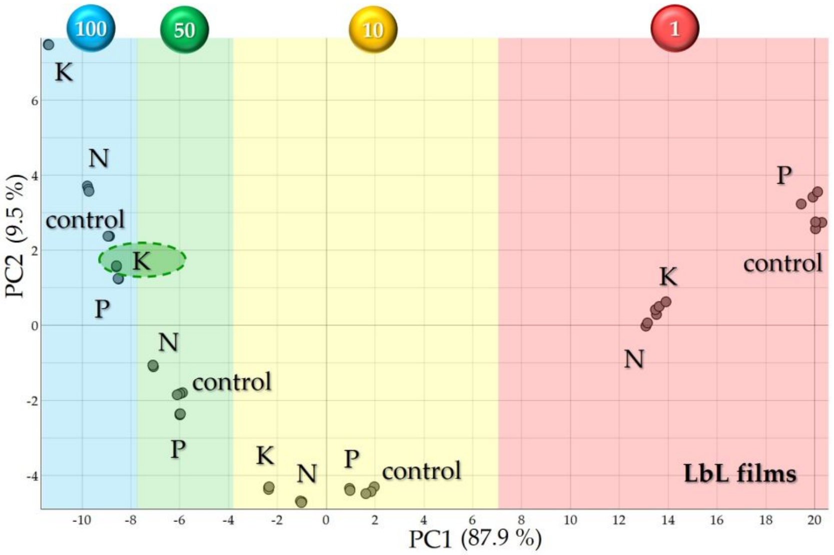

Figure S6: PCA score plots of the impedance data of control, N, P, and K soil samples dispersed in water at 1 (red), 10 (yellow), 50 (green), and 100 (blue) mg/mL obtained with the multisensory array based on LbL films. Colored areas are just a guide to the eye, once soil sample enriched with K at 50 mg/mL is in the blue area merged with the aliquots at 100 mg/mL (highlighted in the green ellipse).

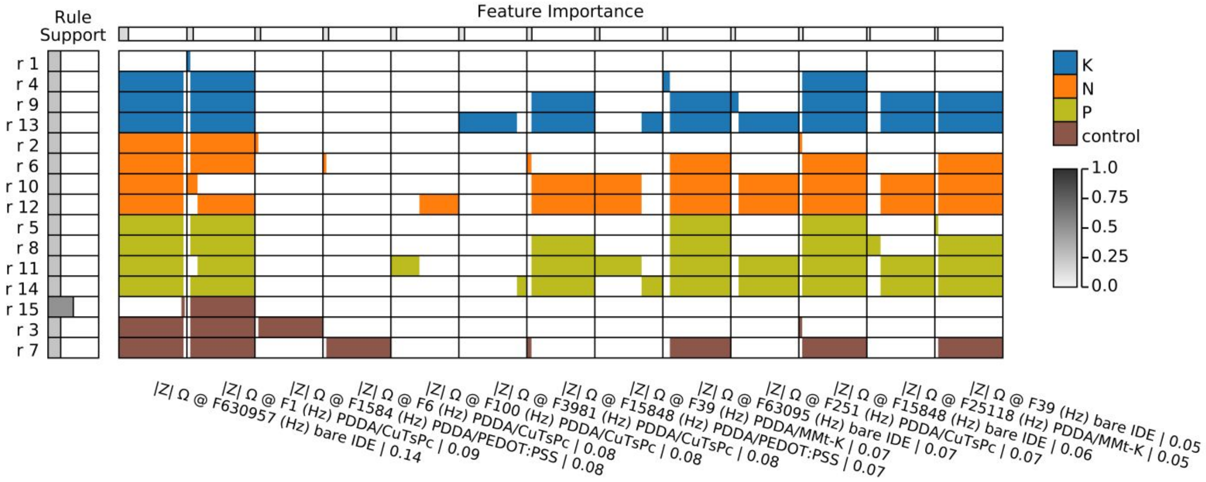

Figure S7: Multidimensional Calibration Space (MCS) for the multisensor array based on LbL films. The space has 13 dimensions corresponding to 13 frequencies (features) selected among the 124 available (F1 to $F10^6$ Hz for 4 sensors). Rules with maximum support are not found, which may indicate a certain data complexity, and at least two frequencies from each sensor employed in the multisensor array (bare IDE, PDDA/CuTsPc, PDDA/MMt-K, and

PDDA/PEDOT:PSS) are used. The average accuracy estimated for the MCS via the DT model is 87%.

References

[1] D.O. Kirsanov, A. V. Legin, A.P. Kulikova, E.N. Pol'shin, Y.G. Vlasov, Polymeric sensors for determination of anions of organic acids, Russ. J. Appl. Chem. 80 (2007) 799–804. https://doi.org/10.1134/S1070427207050205.

[2] Merck, Carbonate Ionophore, (n.d.). https://www.sigmaaldrich.com/RU/en/search/carbonate-ionophore?focus=products&page=1&perpage=30&sort=relevance&term=carbonate ionophore&type=product.

[3] Merck, Tridodecylmethylammonium chloride, (n.d.). https://www.sigmaaldrich.com/FR/en/search/7173-54-8?focus=products&page=1&perpage=30&sort=relevance&term=7173-54-8&type=cas_number.

[4] Merck, Sulfate-ionophore I, (n.d.). https://www.sigmaaldrich.com/RU/en/product/sial/17892.

[5] Merck, 5,10,15,20-Tetraphenyl-21H,23H-porphine manganese(III) chloride, (n.d.). https://www.sigmaaldrich.com/RU/en/search/32195-55-4?focus=products&page=1&perpage=30&sort=relevance&term=32195-55-4&type=cas_number.

[6] Merck, Hydrogen sulfite ionophore I, (n.d.). https://www.sigmaaldrich.com/RU/en/product/sial/54110.

[7] Merck, Potassium tetrakis[3,5-bis(trifluoromethyl)phenyl]borate, (n.d.). https://www.sigmaaldrich.com/RU/en/search/105560-52-9?focus=products&page=1&perpage=30&sort=relevance&term=105560-52-9&type=cas_number.

[8] A.V. Legin, V.A. Babain, D.O. Kirsanov, O.V. Mednova, Cross-sensitive rare earth metal ion sensors based on extraction systems, Sensors Actuators B Chem. 131 (2008) 29–36. https://doi.org/10.1016/j.snb.2007.12.002.
